# Deubiquitylating enzymes and their emerging role in plant biology

**DOI:** 10.3389/fpls.2014.00056

**Published:** 2014-02-19

**Authors:** Erika Isono, Marie-Kristin Nagel

**Affiliations:** Department of Plant Systems Biology, Technische Universität MünchenFreising, Germany

**Keywords:** deubiquitylation, DUB, USP, UBP, UCH, JAMM, OTU, MJD

## Abstract

Ubiquitylation is a reversible post-translational modification that is involved in various cellular pathways and that thereby regulates various aspects of plant biology. For a long time, functional studies of ubiquitylation have focused on the function of ubiquitylating enzymes, especially the E3 ligases, rather than deubiquitylating enzymes (DUBs) or ubiquitin isopeptidases, enzymes that hydrolyze ubiquitin chains. One reason may be the smaller number of DUBs in comparison to E3 ligases, implying the broader substrate specificities of DUBs and the difficulties to identify the direct targets. However, recent studies have revealed that DUBs also actively participate in controlling cellular events and thus play pivotal roles in plant development and growth. DUBs are also essential for processing ubiquitin precursors and are important for recycling ubiquitin molecules from target proteins prior to their degradation and thereby maintaining the free ubiquitin pool in the cell. Here, we will discuss the five different DUB families (USP/UBP, UCH, JAMM, OTU, and MJD) and their known biochemical and physiological roles in plants.

## INTRODUCTION

Post-translational modification through ubiquitin, or ubiquitylation, plays a key role in many aspects of plant development, growth and environmental- as well as immune responses (reviewed in; Vierstra, 2009, 2012). Ubiquitylation must therefore be strictly controlled and regulated at multiple steps during these processes. The attachment of ubiquitin to the target proteins is carried out by the sequential activities of the ubiquitin activating enzyme (E1), ubiquitin conjugating enzymes (E2s), and ubiquitin ligases (E3s) (reviewed in; Hershko and Ciechanover, 1998). The ubiquitylation status of the substrate proteins is also controlled by the activity of deubiquitylating enzymes (DUBs: also deubiquitinating enzymes or deubiquitinases), hydrolases that remove covalently attached ubiquitin molecules from substrates or hydrolyze the peptide bond between ubiquitin molecules. Notably, whereas the *Arabidopsis* genome encodes more than 1500 E3s (Vierstra, 2012), only around 50 DUBs can be identified. This may owe to the fact that in order to deubiquitylate their targets, DUBs may not need direct interaction with the target proteins themselves but rather interact with the ubiquitin chains and hence, DUBs can deal with a broader range of ubiquitylated target proteins.

**FIGURE 1:**
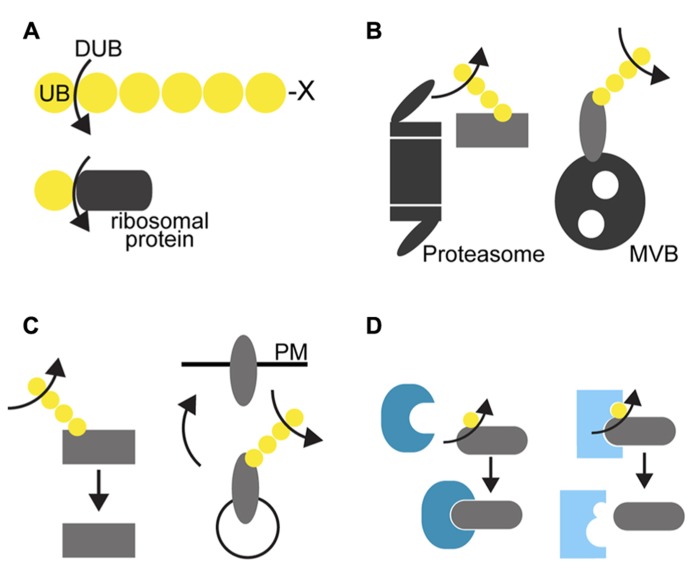
** Cellular function of DUBs. (A)** Ubiquitin is translated as tandem ubiquitin repeats with several amino acid extension (depicted as X) at the C-terminus or as fusion to ribosomal proteins in plants. DUBs process the peptide bond between ubiquitin and its fusion protein to produce ubiquitin monomers that can be then conjugated to its substrate proteins. **(B)** DUBs can remove ubiquitin chains from its target proteins and recycle ubiquitin molecules prior to degradation by the 26S proteasome (left) or before the sequestration into the intraluminal vesicles of the multivesicular body (right). Deubiquitylation can start at the distal end as shown here or at the proximal end or in the interior of polyubiquitin chains. **(C)** Removal of the ubiquitin chains by DUBs can inhibit their recognition by the degradation machinery and thus rescues them from degradation regardless whether the protein is a cytosolic proteasomal substrate (left) or a membrane cargo (right). **(D)** Ubiquitylation can serve as an interaction signal for the modified protein. By removing the ubiquitin moiety, DUBs could change the binding affinity of its target protein to another protein, either by enabling (left) or by disabling the binding of the unmodified protein to its interacting protein.

DUBs have multiple key roles in the regulation of cellular events. Firstly, they are essential for the activation of ubiquitin molecules after translation. Ubiquitin is translated either as tandem linear ubiquitin repeats or fusion to ribosomal proteins in *Arabidopsis* (Callis et al., 1990, 1995) and has to be processed to single ubiquitin molecules by DUBs in order to be conjugated to their substrates (**Figure 1A**). Secondly, they are responsible for the recycling of the ubiquitin molecules by cleaving them off from the substrates prior their degradation either by the 26S proteasome or by vacuolar proteases (**Figure 1B**). In this way, DUBs contribute to maintain the free ubiquitin pool in the cell. Thirdly, DUBs can also actively regulate cellular processes by influencing the stability of proteins, in that they rescue proteins from degradation by deubiquitylating them before they are recognized by the degradation machinery (**Figure 1C**). Finally, by removing the ubiquitin molecule from its target, DUBs could affect the binding affinity of the target protein to its interactor protein and thereby regulate downstream processes (**Figure 1D**).

In eukaryotes, there are five DUB families that can be classified according to the difference in their catalytic domains [Reviewed in (Komander et al., 2009; Reyes-Turcu et al., 2009)]: the ubiquitin-specific proteases (UBPs or USPs), the ubiquitin C-terminal hydrolases (UCHs), the ovarian tumor proteases (OTUs), the Machado–Joseph domain (MJD)- or Josephine domain proteases and the JAB1/MPN/MOV34 (JAMM) proteases. All DUBs are cysteine proteases, except DUBs of the JAMM family, which are zinc metalloproteases that require a coordinated Zinc ion in their active sites. Some of the DUBs display also hydrolysis activity toward other ubiquitin-like proteins, like Nedd8/RUB (Kumar et al., 1993; Callis et al., 1995; Hochstrasser, 1996), SUMO (Matunis et al., 1996), or ISG15/UCRP (Loeb and Haas, 1992), suggesting a complex regulatory mechanism surrounding ubiquitin- and ubiquitin-like modifications.

In most of the cases, interaction of DUBs with their target proteins is mediated outside of the catalytic domain by scaffold proteins or adaptor proteins whereas structural characteristics of the catalytic domain mediate the specificity toward certain ubiquitin linkages (reviewed in; Komander et al., 2009). The structure of the catalytic domains also determines whether the DUB cleaves ubiquitin chains from the distal or the proximal end. Only a few cases were reported in which the DUB were shown to interact directly with its ubiquitylated substrate protein (reviewed in; Reyes-Turcu et al., 2009). In addition, DUB activity can be regulated at the transcriptional or post-translational level in that the DUBs themselves can be phosphorylated, SUMOylated or ubiquitylated (reviewed in; Huang and Cochran, 2013). Thus, it is difficult to associate a specific DUB family to one cellular process, rather, the biochemical and physiological function of each DUB has to be examined individually.

As summarized in the following sections, accumulating evidence indicate an important role of DUBs not only in yeast and mammals but also in various aspects of plant biology. However, in contrast to target protein regulation by the ubiquitylation machinery, understanding of the molecular mechanisms of cellular and physiological functions of DUBs in plants has just started.

## DUB FUNCTION IN PLANTS

### UBIQUITIN-BINDING PROTEINS

**FIGURE 2:**
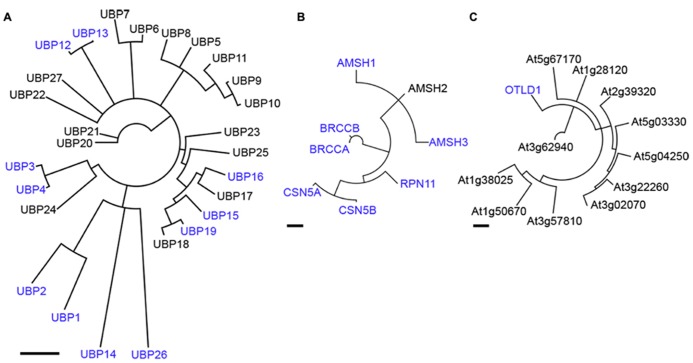
** Phylogenetic analyses of *Arabidopsis* UBP-, JAMM-, and OTU-domain proteinases**. A Neighbor-Joining consensus tree based on amino acid sequences surrounding the catalytic domain for *Arabidopsis* UBP- **(A)**, JAMM- **(B)** and OTU- **(C)** domain proteins is shown. Scale bars indicate 0.5 aa substitutions per site. DUBs mentioned in the text are highlighted in blue. Note that CSN5A and CSN5B shown in **(B)** are deneddylating- and not deubiquitylating enzymes.

The UBPs form the largest subfamily of cysteine protease DUBs in *Arabidopsis* with 27 members that can be classified in 14 subfamilies based on their domain organization (Yan et al., 2000; **Figure 2A**). Most of the UBPs have additionally to their catalytic domain further domains that enable them to interact with different proteins, allowing UBPs to be involved in a broad range of biological processes. However, to date, the molecular functions of UBPs are far from being well resolved in plants, UBP26 being the only member among this family for which the target protein, Histone H2B, is identified (Sridhar et al., 2007).

UBP1 and UBP2 are close homologs to each other and are unique to plants (Yan et al., 2000). They are both active DUBs that can hydrolyze K48-linked**ubiquitin chains *in vitro*. T-DNA insertion mutants *ubp1* and *ubp2* are phenotypically indistinguishable from wild type plants under normal conditions or standard stress conditions that were tested for known proteasomal mutants. However, when grown in the presence of the Arg analog canavanine, mutants show stunted growth, shorter roots and display chlorotic leaves, indicating that UBP1 and UBP2 are necessary for resistance to canavanine.

UBP3 and UBP4 are highly homologous to each other (Doelling et al., 2007). *ubp3* and *ubp4* single mutants do not show obvious phenotypes whereas the double mutant *ubp3ubp4* shows lethality, indicating redundant functions between UBP3 and UBP4. UBP3 and UBP4 are probably required for pollen transmission since *ubp3ubp4 *is defective in gametogenesis and shows also pollen germination defects.

UBP12 and UBP13 show activity toward K48-linked diubiquitin (Ewan et al., 2011). The *ubp12ubp13 *double mutant, but not the single mutants*,* showed lethality, indicating redundant functions between *UBP12* and *UBP13.* *UBP12* and *UBP13* were identified as genes that were up-regulated in response to *Pst* DC3000 infection (Brazma et al., 2003). Accordingly, RNAi line that has reduced levels of both *UBP12* and *UBP13* shows increased disease resistant upon* P. syringae *infection. These results indicated that both UBP12 and UBP13 act as negative regulators in *Arabidopsis* immune response.

*Arabidopsis* UBP14 is a functional homolog of the yeast Ubp14p and is a ubiquitously expressed DUB that cleaves K48-linked chains and Ub-X-βgal, but not UBQ1 (Doelling et al., 2001). The *ubp14* mutant arrested growth during embryo development. The arrested embryos accumulated high amount of ubiquitylated proteins, indicating that UBP14 is an essential DUB, required for proper embryogenesis. Interestingly, UBP14 was also identified as the causative gene of an EMS mutant *phosphate deficiency root hair defective 1* (*per1*) which is defective in Pi deficiency-induced root hair formation (Li et al., 2010). *per1* shows reduced levels of UBP14/PER1 protein, and failed to respond to Pi starvation by increasing the frequency and length of root hairs, implicating UBP14/PER1 function also in the adaptation to changes in phosphate/nutrient availability in the environment.

UBP15 can cleave peptide bonds between tandem ubiquitin and localizes both to the cytosol and the nucleus (Liu et al., 2008). *UBP15* is mainly expressed in leaves, which is in accordance to its proposed function in defining leaf pattern and shape of the leaf margin by controlling cell proliferation. Many genes including cell cycle or flowering genes are misregulated in the *ubp15* mutant, which may be the cause for the developmental defects observed in this mutant. Further genetic analysis has suggested that UBP15 and UBP16 might function redundantly. The *ubp19* mutant was described as embyo-lethal, but no further analysis is yet performed.

*UBP26/SUP32* was first identified in a suppressor screen of *ros1-1*, a mutant with enhanced gene silencing (Sridhar et al., 2007). UBP26 can deubiquitylate monoubiquitylated histone H2B *in vitro*, and the *ubp26-1* mutant accumulates ubiquitylated histone H2B. Further experiments suggested that histone H2B deubiquitylation by UBP26 is important for heterochromatic histone H3 methylation and DNA methylation and hence, for proper gene silencing. Further studies have identified the MADS-box gene *PHERES1* to be probably under this regulation. A T-DNA insertion line of *UBP26* arrested growth at the embryo stage, probably due to the misregulation of *PHERES1* that is normally under the strict regulation of genomic imprinting (Luo et al., 2008).

The *ubp26-1* mutant also shows misregulation of the *FLOWERING LOCUS C* (*FLC)* gene, which leads to an early flowering phenotype of the mutant (Schmitz et al., 2009). Expression of *FLC* is decreased in the *ubp26-1* mutant and ubiquitylated H2B was observed to accumulate in the *FLC* chromatin. Deubiquitylation of H2B by UBP26 probably keeps the levels of H3K27me3 low, thereby allowing activation of *FLC* gene expression. UBP26, together with OTLD1 that is mentioned below, are examples in which DUBs play an active regulatory function that is not directly associated with protein degradation.

### UBIQUITIN C-TERMINAL HYDROLASES

Deubiquitylating enzymes of this family contain a UCH domain, first identified in the yeast Uch1p, which has a structural feature distinct from other DUBs. Mutational studies based on human UCH proteins have revealed a size-filtering mechanism that allows UCH proteins to hydrolyze small ubiquitin adducts more efficiently than ubiquitin chains or large ubiquitin fusion proteins (Popp et al., 2009). For this specificity, UCH proteins are thought to be mainly involved in ubiquitin recycling rather than regulating substrate proteins through deubiquitylation, though several mammalian studies also indicate regulatory roles for UCH family DUBs.

In *Arabidopsis*, three UCH domain proteins were identified and characterized (Yang et al., 2007). *Arabidopsis* UCH1 and UCH2 contain a related C-terminal extension of 100 aa that is missing in UCH3. UCH2 was shown to be able to cleave peptide and/or isopeptide bonds bound to ubiquitin and showed activity toward K48 chains *in vitro*. UCH1 and UCH2 are expressed ubiquitously and GFP-fusion proteins of UCH1 and UCH2 are localized to the nucleus like the 26S proteasome, however, stable association with the proteasome could not be demonstrated. Both UCH1 overexpressing plants as well as a *uch1uch2* double mutant show a number of developmental phenotypes including altered sensitivity to auxin and cytokinins. Moreover, auxin signaling mutants *axr1-3* and *axr2* show both synergy with UCH1 overexpressing lines and in accordance with this, stability of AUX/IAA proteins were found to be specifically modified in the UCH1 overexpressor and *uch1-1uch2-1* double mutant, indicating the involvement of UCH proteins in the auxin signaling pathway.

### JAMM DOMAIN PROTEINS

The JAMM domain DUBs are zinc metalloproteases that contain a catalytic MPN+ domain (Maytal-Kivity et al., 2002). The MPN+ domain coordinates two zinc ions that activate a water molecule to attack the ubiquitin isopeptide bond. One member of the family, human AMSH-Like protease (AMSH-LP), was the first DUB that was co-crystalized with diubiquitin and structural studies provided insightful information regarding the K63-specificity of this DUB (Sato et al., 2008). Eight JAMM domain proteins are present in *Arabidopsis* (**Figure 2B**), most of them being associated with key regulatory roles. CSN5, which is encoded by two homologous genes *CSN5A* and *CSN5B* in *Arabidopsis*, is a JAMM domain protease and the catalytic subunit of the COP9 signalosome that specifically hydrolyzes the ubiquitin-like molecule Nedd8/RUB, rather than ubiquitin (Chamovitz et al., 1996; Cope et al., 2002).

RPN11 was first identified as a subunit of the 26S proteasome regulatory particle in yeast (Glickman et al., 1998) and was subsequently shown to possess deubiquitylating activity (Verma et al., 2002). *Arabidopsis* has one homolog of RPN11, which was shown to be part of the purified *Arabidopsis* 26S proteasome (Book et al., 2010). RPN11 function is primarily required for the deubiquitylation of proteasomal substrates prior to degradation and recycling of ubiquitin molecules.

In contrast to RPN11 and CSN5, AMSH3 is not a stable subunit of a multi protein complex (Isono et al., 2010). It is an essential DUB in *Arabidopsis*, since the *amsh3* null mutants show seedling lethality and a number of intracellular trafficking defects, implicating its function in this pathway. AMSH1, an AMSH3 homolog, and AMSH3 both interact with ESCRT-III subunits (Katsiarimpa et al., 2011, 2013) and are probably involved in the deubiquitylation of plasma membrane cargos at the multivesicular body. Furthermore, mutants of both *amsh1* and ESCRT-III show defects in autophagic degradation, indicating that the ESCRT-III- and AMSH-dependent trafficking pathway is also contributing to the regulation of autophagy (Katsiarimpa et al., 2013). 

*Arabidopsis* BRCC36A and BRCC36B are homologs of mammalian BRCC36, a DUB that was shown to interact with a protein complex containing BRCA1 (Dong et al., 2003) and is recruited to the site of DNA damage (Inui et al., 2011). Though *Arabidopsis* *brcc36* mutants are viable and phenotypically indistinguishable from wild-type plants, the *brcc36a* mutant shows defects in intra- and inter-chromosomal homologous recombination as well as in DNA crosslink repair (Block-Schmidt et al., 2011). BRCC36 was also shown to be epistatic to BRCA1, indicating its involvement in BRCA1 regulation, probably as part of a multi protein complex including BRCC36 and BRCA1, as proposed in other organisms.

### OVARIAN TUMOR PROTEASES

Ovarian tumor proteases are cysteine protease DUBs that contain the OTU-domain, which was first identified in the product of the drosophila *ovarian tumor* gene and is found in virus, bacteria and eukaryotic organisms (Kumar et al., 1993). A recent study using structural and enzymatic analyses of OTU proteases have revealed the mechanism of ubiquitin linkage specificity of human OTU DUBs (Mevissen et al., 2013). The *Arabidopsis* genome contains 12 OTU domain-containing genes (**Figure 2C**) most of which are uncharacterized yet. OTUs are involved in a variety of cellular processes in yeast and mammals but in plants so far only one OTU-protein, OTLD1, was characterized in relation to a specific biological process.

OTLD1 is an otubain-like DUB that was found in a yeast two-hybrid screen using the histone demethylase KDM1C as bait (Krichevsky et al., 2011). Thus OTLD1, like UBP26 mentioned above, is implicated in histone deubiquitylation. OTLD1 was shown to bind to histones and possess DUB activity specifically toward ubiquitylated H2B but not toward H2A. In both a KDM1C mutant *swp1-1* and an *otld1* T-DNA insertion mutant, gene derepression was observed, indicating that KDM1C and OTLD1 function together to repress gene expression via histone deubiquitylation.

### MACHADO–JOSEPH DOMAIN

MJD DUBs are named after the chronic degenerative Machado–Joseph disease. In MJD patients, a cysteine proteinase DUB called Ataxin 3 is modified in its poly Q tract, which probably causes alteration in its structure and interaction with other proteins (reviewed in; Costa Mdo and Paulson, 2012). Ataxin 3 contains the catalytic DUB domain named Josephine-domain and is implicated in proteasome-dependent protein quality control. *In silico* search in the *Arabidopsis* genome database shows three Josephine domain-containing proteins (AT1G07300, AT2G29640, and AT3G54130), the function of which has yet to be elucidated.

### FUTURE RESEARCH ON PLANT DUBs

Although many lines of evidence suggest that not only ubiquitylating enzymes but also DUBs can actively regulate substrate fate, elucidation of the molecular function of individual DUBs in plants has just begun. Studies in the past decade, mainly conducted using yeast and mammalian models, have shown important house keeping- as well as diverse regulatory functions of DUBs in different pathways.

DUBs like the yeast Doa4p (Dupre and Haguenauer-Tsapis, 2001), human and plant AMSH proteins (McCullough et al., 2004; Isono et al., 2010; Katsiarimpa et al., 2011) and human USP8/USPY (Mizuno et al., 2006; Row et al., 2006) were shown to be involved in the regulation of cargo endocytosis and stability. However, it is still an open question whether ubiquitylated endocytosis cargos are direct targets of these DUBs.

DUBs regulate their substrates not only by determining their proteolytic fate. For example, as it was also shown in plants, histone H2A or histone H2B ubiquitylation status is controlled by multiple DUBs (Joo et al., 2007; Sridhar et al., 2007; Zhu et al., 2007; Nakagawa et al., 2008; Schmitz et al., 2009). The ubiquitylation status of histones affects their methylation status and thus controls gene expression in the corresponding chromatin region. In TGFβ signaling, two human DUBs, USP9x, and USP15, were shown to control monoubiquitylination of their substrates Smad4 and R-SMADs, respectively. The ubiquitylation status of these proteins affects their DNA-binding capacity and hence downstream gene activation (Dupont et al., 2009; Inui et al., 2011). It is an intriguing future topic whether plant DUBs are also part of the conserved or plant-specific signaling cascades.

As these examples show, in addition to the understanding of the spatio-temporal regulation of DUBs themselves, the identification of DUB substrates is crucial for the elucidation of individual DUB function. Since structural studies indicate that in most of the studied cases the interaction of DUBs with the ubiquitin chain, but not with their specific target proteins, is the prerequisite for deubiquitylation, the identification of *bona fide* DUB targets is not an easy task. With the advance in quantitative proteomics coupled with the use of suitable mutants and biochemical tools, it is to be expected that we will get a better insight into plant DUB targets in the near future. Further studies should reveal the sophisticated balancing mechanisms of ubiquitylation and deubiquitylation by which substrate fate and thus important intracellular and physiological processes in plants are regulated.

## Conflict of Interest Statement

The authors declare that the research was conducted in the absence of any commercial or financial relationships that could be construed as a potential conflict of interest.
